# Prophylactic Antibiotics for Urinary Tract Infections after Urodynamic Studies: A Meta-Analysis

**DOI:** 10.1155/2021/6661588

**Published:** 2021-02-28

**Authors:** Xiao-yu Wu, Yu Cheng, Sheng-fei Xu, Qing Ling, Xiao-yi Yuan, Guang-hui Du

**Affiliations:** Department of Urology, Tongji Hospital, Tongji Medical College, Huazhong University of Science and Technology, Wuhan, Hubei Province, China

## Abstract

**Aim:**

We aimed to perform a meta-analysis to determine whether antibiotic prophylaxis reduces the incidence of urinary tract infections (UTIs) after urodynamic studies (UDS).

**Methods:**

We conducted a systematic search of PubMed, Web of Science, Ovid, Elsevier, ClinicalKey, Embase, Cochrane Library, Medline, and Wiley Online Library. Randomized controlled trials (RCTs) comparing the effectiveness of prophylactic antibiotics with placebo or no treatment in preventing UTI after UDS were included. Two reviewers extracted data independently, and RevMan 5.3 software was used to analyze relative risk (RR) with 95% confidence intervals (CI). Heterogeneity was assessed by the *Q* test and *I*^2^ test.

**Results:**

The final meta-analysis included 1829 patients in 13 RCTs. Compared with the placebo or no treatment group, prophylactic antibiotics could significantly reduce the risk of bacteriuria (RR = 0.42, 95% CI: 0.30-0.60) and the risk of symptomatic UTI (RR = 0.65, 95% CI: 0.48-0.88). In addition, there was no statistically significant difference in the risk of adverse events (RR = 4.93, 95% CI: 0.61-40.05). No significant heterogeneity or publication bias was found in this study.

**Conclusions:**

Current evidence showed that prophylactic antibiotics could reduce the risk of asymptomatic bacteriuria and symptomatic UTI after UDS without increasing the incidence of adverse events.

## 1. Introduction

Urodynamic studies (UDS) are widely used in gynecology, urology, geriatrics, pediatrics, and rehabilitation [1]. These are common and tolerable tests used to investigate lower urinary tract and pelvic floor dysfunction, such as neurogenic bladder, urinary incontinence, and voiding dysfunction [2]. In general, UDS is divided into two types that include a noninvasive test called free flowmetry and an invasive test called filling cystometry and pressure-flow study. When invasive UDS is performed, a catheterization will be inserted from the external urethral orifice into the bladder to pump water or contrast media into the bladder and record pressure, which can cause tissue damage and introduction of external pathogens and may also generate discomfort, anxiety, pain, distress, hematuria, and urinary tract infection (UTI) for patients [1, 3–5].

UTI is often classified into asymptomatic bacteriuria (ABU) and symptomatic UTI. ABU is defined as positive urine culture, the asymptomatic carriage of >10^5^ bacteria/mL in urine specimen, with or without pyuria, in two consecutive cultures [6, 7]. Establishing a diagnosis of symptomatic UTI requires a patient to have laboratory tests confirming the presence of bacteriuria (bacteriuria > 10^5^ CFU/mL and pyuria > 10 white blood cells/high powered field) and symptoms and signs of a UTI, such as fever, worsened urinary urgency or frequency, dysuria, suprapubic tenderness, costovertebral angle pain, tenderness, blood while passing urine, and more smell than typical smell of urine with no recognized cause [6, 7]. Among the patients who undergo invasive UDS, UTI is the most studied complication. Although the perineum is disinfected before the urodynamic examination, patients still suffer from UTI. The incidence of acquired UTI (asymptomatic or symptomatic) after urodynamic examination ranges from 1.5 to 36% [6–9]. This is a wide discrepancy and may be due to various factors, such as the time of urine testing, catheterization technique [10], the difference in study populations in terms of age or underlying problems, UDS performance method, and different definitions of urinary tract infections [11, 12].

Prophylactic antibiotics are often used in several urologic invasive treatments such as catheter insertion and UDS to prevent UTI posttreatment. However, previous studies regarding the use of prophylactic antibiotic post-UDS came up with inconsistent results. To prevent UTI resulting from invasive UDS, antibiotic prophylaxis is routinely administered before or immediately after a urodynamic test in many medical institutions [9, 13], whereas other studies have suggested antibiotic prophylaxis was useless [14, 15]. Although the results from different studies were conflicting, two previous meta-analyses reported that the use of prophylactic antibiotics reduced the bacteriuria risk caused by urodynamic tests [6, 7]. Since the bacteriuria sometimes was asymptomatic and self-limited, we should pay more attention to symptomatic UTI. Coptcoat et al. [15] concluded that prophylactic antibiotic could not reduce posturodynamic investigation irritative symptoms. In another study, no patients who received ciprofloxacin developed symptomatic UTI, while 14% of patients suffered in the placebo group [16]. However, the protective efficacy of antibiotic prophylaxis did not attain statistical significance. In addition, Rahardjo et al. [17] found that a three-day course of levofloxacin of 500 mg daily could decrease the incidence of symptomatic UTI from 28.6% to 12.7%. However, there is not a high level of evidence suggesting that the risk of symptomatic UTI can be reduced after using prophylactic antibiotics. Thus, in the present study, we performed a meta-analysis to determine whether antibiotic prophylaxis can reduce the incidence of bacteriuria risk and symptomatic UTI after UDS.

## 2. Materials and Methods

This meta-analysis was performed in accordance with PRISMA guidelines.

### 2.1. Search Strategy

We conducted a systematic search of PubMed, Web of Science, Ovid, Elsevier, ClinicalKey, Embase, Cochrane Library, Medline, and Wiley Online Library. The following search terms were used: (“antibiotic” or “antibiotics”) and (“urodynamic” or “urodynamics”). We also searched the references of the retrieved literatures for additional studies. There were no restrictions on the time period, sample size, population, or language. If more than one article was published using the same case series, only the study with the largest sample size was included.

### 2.2. Inclusion and Exclusion Criteria

The included studies in this meta-analysis met the following criteria: (a) RCTs comparing the use of antibiotics versus placebo or no antibiotics and (b) the main outcome indicators included at least the incidence of bacteriuria, or the incidence of symptomatic UTI. The exclusion criteria were as follows: (a) repeated publications, (b) studies with incomplete information and data that could not be extracted, and (c) review, popular science, and opinion literature.

### 2.3. Data Extraction

Two investigators (X.W. and S.X.) independently extracted the data from all of the eligible publications according to the inclusion and exclusion criteria. Any disagreement was resolved by discussion with another investigator. The following information was extracted from each study: first author's name, year of publication, study location, sample size, gender, type, dose and duration of test drug, adverse events, etc.

### 2.4. Quality Assessment

The quality assessment was based on the Cochrane systematic review manual, including ① selection bias (generation and allocation concealment of random sequences), ② implementation bias (blinding researchers and subjects), ③ measurement bias (blindness of study result evaluation); ④ follow-up bias (completeness of result data), ⑤ report bias (selective report of study results), and ⑥ other bias (other sources of bias).

### 2.5. Statistical Analysis

All statistical analyses were performed using RevMan 5.3 (Cochrane Library Software, Oxford, UK). Relative risk (RR) and its 95% confidence interval (CI) were used to analyze the data. Heterogeneity among the included studies was checked by Cochrane's *Q* test and *I*^2^ test. If the data showed little heterogeneity (*P* ≥ 0.1 and *I*^2^ < 50%), a fixed-effect model was used; otherwise, a random-effects model was used. Publication bias was assessed by funnel plot, Begg's test, and Egger's test. *P* < 0.05 was considered as statistically significant.

## 3. Results

### 3.1. Search Results

**Figure 1:**
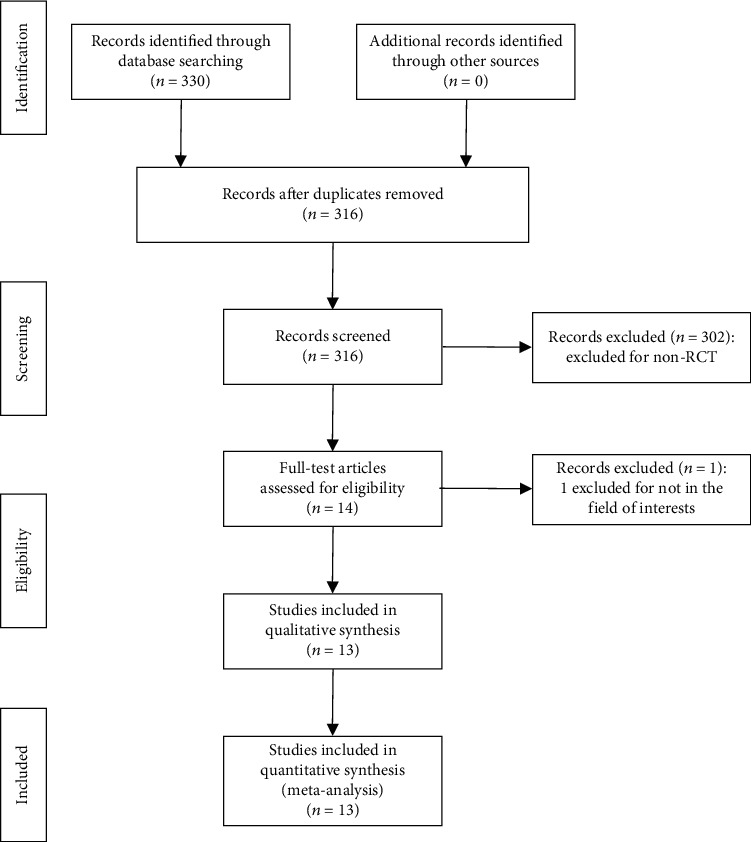
Flow diagram of the study search and selection.

A total of 330 articles were identified from the major databases described above. After removing 14 duplicate publications, 302 non-RCT studies were excluded by reading titles and abstracts, and 1 article was excluded for cystoscopy. Finally, 13 articles were included, including 2 abstracts. The literature selection process is shown in Figure 1. The basic characteristics of the included studies are shown in Table 1.

### 3.2. Quality Assessment

**Figure 2:**
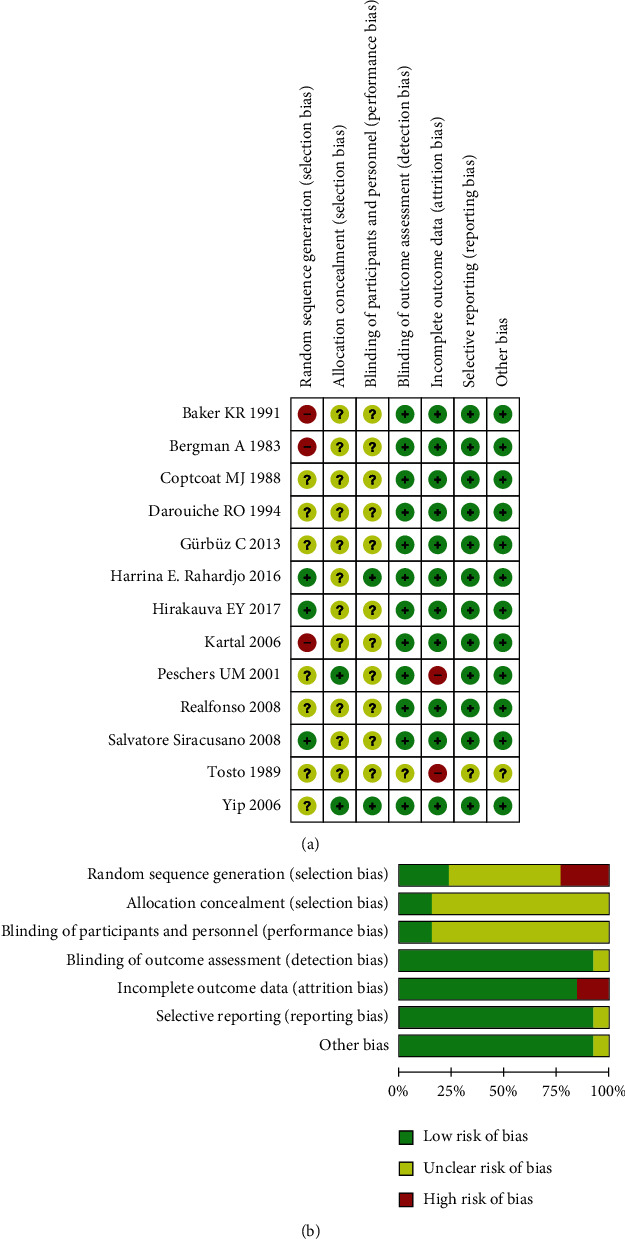
Risk of bias assessment.

Figure 2 shows the risk of bias. Three studies used a reasonable random number generator, seven studies only mentioned “random,” without describing random methods in detail, and three studies used nonrandom methods. Two articles applied allocation concealment, and the others were not clear, which was the same as double-blind, twelve articles were blinded to the outcome assessment, and two articles did not offer complete data. Regarding selective reporting and other biases, twelve articles were at a low risk. All documents reported the outcome data in detail.

### 3.3. Bacteriuria

**Figure 3:**
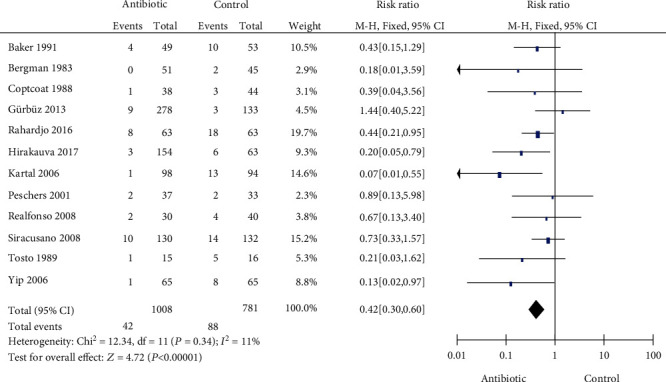
Forest plot for bacteriuria.

Twelve articles, with 1789 patients, were included to assess the effect of prophylactic antibiotics on bacteriuria after UDS. The heterogeneity test (*P* = 0.34 and *I*^2^ = 11%) indicated that the included subgroup studies were homogeneous. The results showed that the prophylactic antibiotics can significantly reduce the risk of bacteriuria after UDS (RR = 0.42, 95% CI: 0.30-0.60) (Figure 3).

### 3.4. Subgroup Analysis

The results showed that the ciprofloxacin, levofloxacin, and amoxicillin-clavulanic acid can significantly reduce the risk of bacteriuria after UDS. A summary of results from all comparisons is listed in Table 2.

### 3.5. Symptomatic UTI

**Figure 4:**
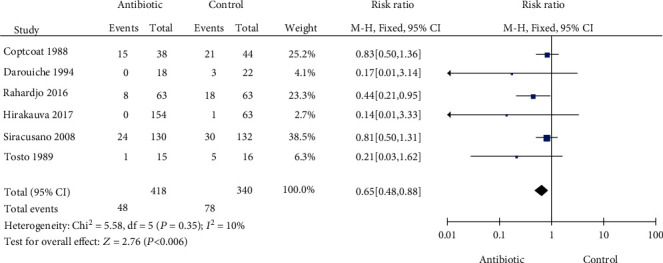
Forest plot for symptomatic UTI.

Six articles with 758 patients were included to assess the effect of prophylactic antibiotics on symptomatic UTI after UDS. The heterogeneity test (*P* = 0.35 and *I*^2^ = 10%) indicated that multiple studies were homogeneous. Prophylactic antibiotics could significantly reduce the risk of symptomatic UTI after UDS (RR = 0.65, 95% CI: 0.48-0.88), see Figure 4.

### 3.6. Side Effects

**Figure 5:**
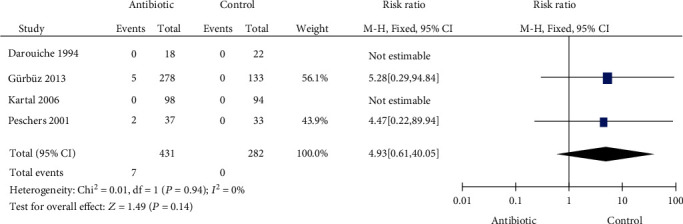
Forest plot of side effects.

Four articles with 713 patients were included to assess the side effects of prophylactic antibiotic use after UDS. The heterogeneity test (*P* = 0.94 and *I*^2^ = 0%) indicated that multiple studies were homogeneous. Our results showed that prophylactic antibiotics did not increase the incidence of drug-related side effects (RR = 4.93, 95% CI: 0.61-40.05) (Figure 5).

### 3.7. Sensitivity and Publication Bias Assessment

**Figure 6:**
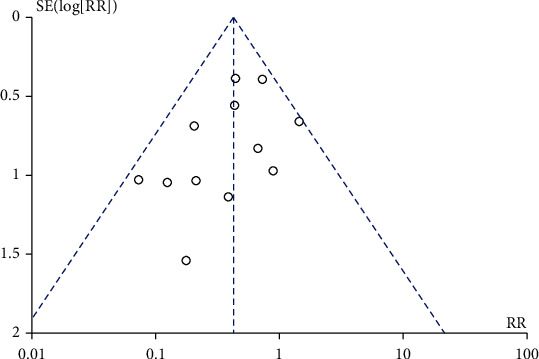
Funnel plot for bacteriuria.

We compared the differences in RR and 95% CI before and after omitting individual studies one by one. The results revealed that there was no substantial change before or after the elimination. The combined value was statistically significant, which indicated that the results of the meta-analysis were robust. The graphs of the twelve studies for bacteriuria were mostly symmetrical in the funnel chart, and all points were mainly concentrated in the middle (Figure 6). According to the results of Egger's (*P* = 0.59) and Begg's test (*P* = 0.21), no significant publication bias was suggested.

## 4. Discussion

Whether prophylactic antibiotics are associated with a reduced risk of symptomatic UTI is still attracting attention from researchers. To date, no meta-analysis has been performed regarding prophylactic antibiotics on UDS-related symptomatic UTI. To the best of our knowledge, the present study was the first meta-analysis to investigate whether antibiotic prophylaxis could reduce the incidence of symptomatic UTI after UDS. The present meta-analysis included a total of 13 RCTs with 1026 patients receiving antibiotics and 803 patients receiving placebo or no treatment. These trials were conducted in 9 regions (Brazil, Indonesia, Turkey, Italy, Hong Kong, Germany, USA, Canada, and British). We performed comparisons of symptomatic UTI in this meta-analysis, while only asymptomatic bacteriuria was compared in the previous meta-analysis [6, 7]. The conclusion of our meta-analysis was that prophylactic antibiotics could reduce the risk of significant asymptomatic bacteriuria and symptomatic UTI, but the incidence of adverse events did not increase.

Previous studies reported that the consequences of UDS-related bacteriuria were most often asymptomatic and transient. In most patients, UTI often manifests in the form of cystitis, which can be easily recognized and treated with drinking water or oral antibiotics [12]. Only a small percentage of patients develop acute pyelonephritis or severe systemic infection [1]. However, irritative symptoms, such as vulval soreness, urinary frequency, and urgency, usually occur after a urodynamic study [10]. Bombieri et al. [10] reported symptoms in 34% of patients after UDS. Quek and Tay [18] reported irritative symptoms in 25% of their subjects after UDS.

To investigate whether prophylactic antibiotics can reduce symptomatic UTI, 6 eligible randomized controlled trials with 758 patients were included for analysis. When compared with placebo or no treatment, the administration of prophylactic antibiotics significantly reduced the risk of symptomatic UTI after urodynamic examination (RR = 0.65, 95% CI: 0.48-0.88). Different rates of symptomatic UTI after UDS in patients who did not use antibiotics have been reported (1.6 to 28.6%), which may be related to the following three factors. First, researchers had different definitions of symptomatic UTI after UDS. Some symptoms were not specific, such as pain, hematuria, dysuria, and frequency. These symptoms may not be all caused by UTI, and mechanical damage by catheters cannot be ruled out [11, 15]. Second, the follow-up of the above studies was also different. Follow-ups were performed 2 weeks [19], 4 days [20], and 3-5 days [16] after urodynamic examination. Third, for the difference in study populations in terms of underlying problems, the subjects in a study by Darouiche et al. [16] were patients with neurogenic lower urinary tract function. The prevalence of symptomatic UTI after UDS in these patients was higher than that in other patients due to bladder dysfunction [21]. It appears that the wide discrepancy in the incidence of symptomatic UTI is because of the above three factors. Therefore, future RCT studies need to be further in accordance in terms of patient selection, definition of symptomatic UTI, and follow-up time.

Twelve articles with 1789 patients were included to assess the effect of prophylactic antibiotics on bacteriuria after UDS. The results showed that prophylactic antibiotics can significantly reduce the risk of bacteriuria after UDS (RR = 0.42, 95% CI: 0.30-0.60), which was consistent with the results of Foon et al. [6, 7]. Different rates of acquired asymptomatic bacteriuria after UDS in patients who did not use antibiotics have been reported (2.3 to 31.3%). In these studies, the incidence of acquired bacteriuria exceeded 10% in 58% (7/12) of studies. Previous literature reports suggest that if the incidence of acquired bacteriuria is greater than 10%, prophylactic antibiotics are recommended [22]. In addition, advanced age, recurrent UTI, previous urologic surgery [1], hypothyroidism, advanced pelvic organ prolapse, BMI > 30 [12], and PVR > 50 mL [11] were independent risk factors for UTI caused by urodynamic examination, which suggested that prophylactic antibiotics should be given to high-risk patients.

For subgroup analyses examining the effect of antibiotic species on bacteriuria, the results showed that the ciprofloxacin, levofloxacin, and amoxicillin-clavulanic acid had a good preventive effect on bacteriuria, which was the same as the previous study [23]. Due to the small number of RCTs, this meta-analysis did not perform subgroup analysis based on different antibiotics with the symptomatic UTI.

The quality of the included randomized controlled trials was poor, which can reduce the reliability of the results. Several studies failed to adopt random sequence generation or failed to offer complete data, and some types of bias of the enrolled studies were unclear, which may weaken the persuasiveness of the evidence. One study indicated that the effect of treatment would be exaggerated by 41% for inadequately concealed trials, 30% for unclearly concealed trials, and 17% for not doubled-blind trials [24]. Another study elaborated that reporting randomized studies and blinding were less likely to report positive findings than those that did not [25]. Therefore, the results of low-quality studies should be interpreted with caution. Unfortunately, no trial was low risk for all the criteria considered. It is worth noting that our search did not include data in languages other than English and Italian, which may result in certain selective bias. The funnel plot and Begger's and Egger's tests did not show significant publication bias because both the positive studies and negative studies were present. Therefore, there was a low risk of publication bias. In the present study, the heterogeneity test showed that multiple studies were homogeneous when we discussed different outcome indicators. The result of the sensitivity analysis, which was performed by omitting a single study in turn, showed no substantial change in the results, indicating good robustness of the meta-analysis results.

## 5. Limitations

Although this study conducted an analysis on the basis of comprehensive literature retrieval and strict inclusion, there were several limitations. First, to minimize selection bias, we included as many articles as possible without exclusion of those too old articles. In the present study, most of the literatures selected were published ten years ago, which may have influenced the results of our analyses. Thus, studies with larger sample sizes and high-quality RCTs are still needed in the future to guide clinical treatment. Second, the methodological quality of the studies included in this study was poor. Some of the included studies lacked randomized sequence generation or failed to offer complete data, and the risk of some bias types of some enrolled studies was unclear. Third, this study only selected English studies and one Italian study, and some related studies published in other languages might have been missed in our meta-analysis. Therefore, the influence of selective bias in the present analysis could not be completely excluded.

## 6. Conclusions

Current evidence showed that prophylactic antibiotics could reduce the risk of asymptomatic bacteriuria and symptomatic UTI after UDS without increasing the incidence of adverse events. This study can help to deliver data of empirical antibiotic therapy during UDS examinations for urologists. However, the prudent use of antibiotics still needs to be emphasized due to the continued increase of the antimicrobial resistance of pathogens worldwide. Prophylactic antibiotics need to be selected according to the regional and local resistance data. In addition, studies with larger sample sizes and high-quality RCTs are still needed in the future to guide clinical treatment.

## Figures and Tables

**Table 1:** Characteristics of selected literature.

First author, year	Gender	Country	Patients (*n*)	Control group	Study group	Period (day)	Follow-up (day)
Hirakauva et al. [19], 2017	F	Brazil	217	Placebo qd (*n* = 63)	Levofloxacin 500 mg qd (*n* = 59), sulfamethoxazole 400 mg-trimethoprim 80 mg qd (*n* = 48), or nitrofurantoin 100 mg qd (*n* = 47)	1 d	14 d
Rahardjo et al. [17], 2016	F/M	Indonesia	126	Placebo qd (*n* = 63)	Levofloxacin 500 mg qd (*n* = 63)	3 d	4 d
Gürbüz et al. [20], 2013	F/M	Turkey	411	Blank control (*n* = 133)	Ciprofloxacin 500 mg qd (*n* = 141), or fosfomycin trometamol unknown dose qd (*n* = 137)	1 d	5-7 d
Siracusano et al. [26], 2008	F	Italy	262	Placebo qd (*n* = 132)	Norfloxacin 400 mg qd (*n* = 130)	1 d	3 d
Realfonso et al. [27], 2008	F	Italy	70	Placebo qd (*n* = 30)/blank control (*n* = 10)	Levofloxacin 500 mg qd (*n* = 30)	1 d	7 d
Kartal et al. [28], 2006	F/M	Turkey	192	Blank control (*n* = 94)	Ciprofloxacin 500 mg qd (*n* = 98)	1 d	2-3 d
Yip et al. [29], 2006	F	Hong Kong	130	Placebo (*n* = 65)	Amoxicillin 250 mg-clavulanic acid 125 mg qd (*n* = 65)	1 d	2 d
Peschers et al. [30], 2001	F	Germany	70	Placebo (*n* = 33)	Trimethoprim 320 mg-sulfamethoxazole 1600 mg qd (*n* = 37)	1 d	14 d
Darouiche et al. [16], 1994	F/M	USA	40	Placebo qd (*n* = 22)	Ciprofloxacin 500 mg bid (*n* = 18)	2 d	3-5 d
Baker et al. [14], 1991	F	Canada	102	Finapyridine 200 mg tid (*n* = 53);	Nitrofurantoin 50 mg q6h-finapyridine 200 mg tid (*n* = 49)	1 d	2-3 d
Coptcoat et al. [15], 1988	F/M	British	82	Blank control (*n* = 44)	Trimethoprim 200 mg qd (*n* = 38)	1 d	2 d
Tosto et al. [13], 1989	F	Italy	31	Blank control (*n* = 16)	Cinoxacin 500 mg bid (*n* = 15)	5 d	3-7 d
Bergman and McCarthy [9], 1983	F	USA	96	Finapyridine 100 mg tid (*n* = 45)	Nitrofurantoin 50 mg-finapyridine 100 mg tid (*n* = 51)	3 d	7 d

**Table 2:** Subgroup study of antibiotics in bacteriuria.

Antibiotic	Studies (*n*)	Patients (*n*)	Effect estimate RR (95% CI)
Ciprofloxacin	3	506	0.38 (0.16, 0.87)
Levofloxacin	3	318	0.42 (0.22, 0.79)
Norfloxacin	1	262	0.70 (0.30, 1.64)
Cinoxacin	1	31	0.16 (0.02, 1.55)
NF-PNPD	2	198	0.38 (0.14, 1.05)
Trimethoprim	1	82	0.37 (0.04, 3.71)
SMZ-TMP	2	181	0.40 (0.10, 1.58)
FT	1	270	0.97 (0.19, 4.89)
AMX-CA	1	130	0.13 (0.02, 0.97)
Nitrofurantoin	1	110	0.22 (0.03, 1.79)

NF-PNPD: nitrofurantoin-phenazopyridine hydrochloride; SMZ-TMP: sulfamethoxazole-trimethoprim; FT: fosfomycin tromethamine; AMX-CA: amoxicillin-clavulanic acid.

## Data Availability

The data used to support the findings of this study are included within the article.
